# Pea Protein Extraction Assisted by Lactic Fermentation: Impact on Protein Profile and Thermal Properties

**DOI:** 10.3390/foods10030549

**Published:** 2021-03-06

**Authors:** Mehrsa Emkani, Bonastre Oliete, Rémi Saurel

**Affiliations:** Physico-Chimie des Aliments et du Vin, PAM UMR A 02.102, AgroSup Dijon, Université Bourgogne Franche-Comté, F-21000 Dijon, France; mehrsa.emkani@agrosupdijon.fr (M.E.); bonastre.oliete@u-bourgogne.fr (B.O.)

**Keywords:** pea protein, alkaline solubilization/isoelectric precipitation, extraction, lactic acid fermentation, thermal properties

## Abstract

Although pea protein has been widely explored, its consumption is still limited by undesirable sensory characteristics and low solubility. All these properties can be modified during protein extraction process. Besides, previous studies showed that lactic acid bacteria (LAB) have a positive effect on legume protein ingredients in terms of flavor and functional properties. Hence, the objective of this work was to explore an alternative extraction method based on alkaline extraction/isoelectric precipitation (AEIEP) resulting in globulin-rich and residual albumin-rich fractions. Here, the decrease in pH was achieved by lactic fermentation instead of mineral acid addition. Different bacteria strains (*Streptococcus thermophilus*, *Lactobacillus acidophilus* and *Bifidobacterium lactis*) have been used alone or in co-culture, and the results were compared with the usual acidification. The extraction assisted by fermentation led to the increase by 20–30% in protein content/yield of the albumin fraction, meaning that the solubility of the extracted pea protein was increased. This result could be explained by the proteolytic activity of bacteria during lactic fermentation. Therefore, the thermal denaturation properties of the isolated protein fractions measured by differential scanning calorimetry could be mainly ascribed to differences in their polypeptide compositions. In particular, higher denaturation enthalpy in globulin fractions after fermentation compared to AEIEP (~15 J/g protein vs. ~13 J/g protein) revealed the relative enrichment of this fraction in pea legumins; a higher part of 7S globulins seemed to be consumed by lactic acid bacteria.

## 1. Introduction

The popularity of legumes is growing as consumers increasingly demand healthier and novel sources of protein, which can totally or partially replace those from animals [1]. Although animal-based foods might be considered as optimal in essential amino acid composition, their consumption is associated with health problems and environmental issues. Therefore, it is urgent to find alternative sources of protein that are available, cost-effective and more promising for both human health and environment. Legumes are a possible solution that contribute toward sustainable and healthier diet [2]. Among various legume proteins, pea protein is of great interest for its worldwide production, high-quality amino acids and low allergenicity [3]. However, the consumption of pea protein is still limited by its imperfect techno-functional properties, low solubility and low accepted sensory profile [4].

In fact, pea is a great source of protein, approximately 20–30% of total dry seed [5]. It can be consumed either as a part of grain components (e.g., flour milled from grains) or as enriched protein ingredients such as protein concentrate (50–55%) and protein isolate (80–90%) [4,6]. The majority of pea proteins consists of albumins (15–20%) and globulins (60–70%) [7]. These two fractions can be purified, since they have different solubility. Albumins are water soluble, while globulins are salt soluble. The two main proteins of the albumin fraction are PA1 and PA2 belonging to the 2S group [8]. PA1 molecules have a molecular weight of approximately 4–6 kDa [9]. PA2 proteins have a molecular weight of about 24–26 kDa and are thought to associate non-covalently to form homodimers of 48–53 kDa [10]. Both 2S pea albumins have a high sulfur amino acid content compared to globulins [11]. The three abundant pea globulins are legumin, vicilin and convicilin with an isoelectric point (pI) close to pH 4.8. Legumins belong to the 11S group and have a multimeric structure with a high molecular weight of 360–400 kDa. A mature legumin consists of six subunit pairs (~60 kDa) that interact non-covalently. Each of these subunit pairs consists, in turn, of an acidic subunit of ~40 kDa and a basic subunit of ~20 kDa, linked by a single disulfide bridge. Vicilin is a trimer of 150–200 kDa. It lacks cysteine, so it cannot form a disulfide bond. Each monomer (~50 kDa) has two cleavage sites, which make possible the post-translational formation of various polypeptide fragments of about 20 kDa (α), 13 kDa (β) and 12–16 kDa (γ), 30–36 kDa (α + β) and 25–30 kDa (β + γ). Finally, convicilin subunits (~70 kDa) are thought to associate non-covalently in a multimer of 210–280 kDa [12,13]. The variety in structures and conformations of pea proteins affects both their functional and nutritional properties. For instance, vicilin has less compact structure, thus, it is more readily extractable and offers better functional properties than legumin [14]. However, the low content of sulfur amino acids in vicilin make it less interesting from a nutritional point of view [15].

Nutritional, sensory and technological properties of pea proteins can be influenced by the extraction process [16]. That is why food researchers and the industry are constantly trying to develop new cost-effective and safe extraction methods with optimal extractability leading to adequate techno-functional and sensory properties [17]. Indeed, numerous methods have been proposed for the extraction of protein from pea flour including alkaline extraction-isoelectric precipitation (AEIEP), salt extraction-dialysis, micellar precipitation and aqueous extraction (pH > 7) [3]. However, each method might be selected for different protein type, which in turn, influences the final composition and functionality of the protein isolate [17]. AEIEP is a common technique with high yield for producing pea protein isolates in the food industry. The method aims at separate albumins and globulins thanks to differential solubility. The reason is the high solubility of both legumin and vicilin at alkaline pH, but minimal solubility at their pI while the albumin part remains soluble [16]. This process starts with the solubilization of protein by suspending pea flour in water at alkaline pH (~8). It then follows by the isoelectric precipitation step in which the pH of the protein-rich extract is lowered up to ~4.5–4.8 by the addition of mineral acid in order to reach pI of the globulins. Then, the precipitated globulins can be neutralized and dried to produce protein isolate as powder.

Food processing including physical, chemical and biological (i.e., fermentation, germination) process is also determinant in controlling protein composition and properties. Fermentation is a traditional technique that serves as a practical method for food preservation [18,19]. It has been used to enhance the bioaccessibility and bioavailability of nutrients [20,21,22,23,24] or to improve the organoleptic properties [25,26,27,28] and shelf life of various legume proteins. Fermentation consists of modifying food by microorganisms (bacteria, molds and yeasts) that grow and consume part of the substrates and enrich it with the products of their metabolism [29]. However, selection of the right microorganism is necessary, since some microorganisms including yeasts and molds might concern food safety [30]. Lactic acid bacteria (LAB) with the generally recognized as safe (GRAS) status are of great interest in food fermentation [31]. They are known for contributing to the improvement of desired sensory properties and improvement of food’s aroma [32].

LAB have been increasingly used for legume fermentation in the last decade. However, its effect is highly related to the legume type, LAB strain and fermentation conditions [33,34]. Lactic acid fermentation can affect the structure and content of legume protein. This can be attributed to the proteolytic activity of bacteria mechanism during fermentation [28], by which the polypeptide chain is broken down, and new polypeptides with a lower molecular weight are formed [27,35]. The changes in protein conformation and structure alter the functionality and nutritional properties of the final products [36,37]. The LAB species such as *Streptococcus thermophilus*, *Lactobacillus delbrueckii subsp. bulgaricus*, *Lactobacillus acidophilus*, *Lactobacillus helveticus* and *Lactobacillus plantarum* have been frequently reported for their positive effects on the organoleptic properties of legume protein [26]. The development of LAB during pea protein fermentation helps the improvement of aroma and flavor by either reducing the occurrence of compounds responsible for off-flavor or masking undesirable green notes [38]. LAB fermentation is also an effective way for partial or complete degradation of antinutritional factors and improvement of protein bioavailability and digestibility [21,22,32,39].

Taking into account the positive effects of LAB fermentation on the legume properties and the drop in pH due to lactic acid formation, the aim of the present study was to explore an alternative extraction method of pea proteins based on alkaline solubilization/isoelectric precipitation, where the decrease in pH was achieved by lactic fermentation instead of mineral acid addition. Three different commercial LAB strain or starters were selected for their aptitude for acidification and/or their recognized positive effect on legume protein properties: *Streptococcus thermophilus* (S), *Streptococcus thermophilus* + *Lactobacillus acidophilus* (SL) and *Streptococcus thermophilus* + *Lactobacillus acidophilus* + *Bifidobacterium lactis* (SLB). The fermentation-assisted extraction was expected to modify the protein profile of the globulin and the albumin fractions isolated with this process. To evaluate this effect, extraction yield and polypeptide profile of the albumin and globulin isolates were evaluated by comparison to unfermented systems produced by common AEIEP. Free amino group content and thermal properties of the samples were further analyzed to check the denaturation of proteins.

## 2. Materials and Methods

### 2.1. Materials

Pea protein was extracted from smooth yellow peas (*Pisum sativum* L.) flour supplied by COSUCRA (Warcoing, Belgium). The total nitrogen content of pea flour, analyzed by the Kjeldhal method, was 3.66 ± 0.02 wt.% (db), and the dry matter content was 88.03 ± 0.06 wt.%. All the chemical products were of analytical grade and supplied by Honeywell FlukaTM (Gillman, SA, Australia). Commercial lyophilized bacteria strains (DuPont Danisco, France) consisted of *Streptococcus thermophilus* (S) (Choozit^TM^ TA 52 LYO 25 DCU), thermophilic multiple-species culture containing *Streptococcus thermophilus* and *Lactobacillus acidophilus* (SL) (YO-MIX^®^ 101 LYO 100 DCU) and multiple species containing *Streptococcus thermophilus, Lactobacillus acidophilus and Bifidobacterium lactis* (SLB) (YO-MIX 202 FRO 500 DCU).

### 2.2. Pea Protein Extraction and Purification

**Figure 1:**
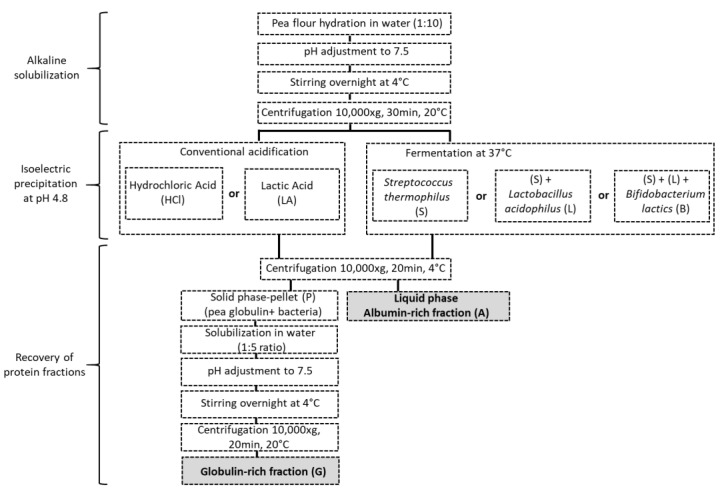
Pea protein extraction method.

The extraction route was indicated in Figure 1. Pea proteins were extracted by AEIEP method, which first comprised an alkaline solubilization of protein and subsequent removal of the insoluble material from pea flour by centrifugation. Hence, pea flour was mixed with water (weight ratio of 1:10), and the pH of the solution was adjusted to 7.5 with NaOH (0.5 M). The solution was then stirred overnight at 4 °C, and pH was readjusted to 7.5. Insoluble material was removed by centrifugation (10,000× *g*, 30 min, 20 °C), and the supernatant was collected. Acid precipitation was used to separate pea globulin-rich and albumin-rich fractions. The acidification was achieved by fermentation with S, SL or SLB starters. The selected strains of bacteria were added into the protein suspensions at usual recommended concentrations indicated by DCU values. The values, in 1.4 L of protein suspension, were 0.119, 0.3 and 0.3 g of S, SL and SLB, respectively. The samples were then placed into an incubator (Sanyo incubator MIR-153 w, Osaka, Japan) at 37 °C under moderate stirring (each fermentation was prepared in triplicate). The pH of the incubated pea protein was measured continuously and automatically recorded at 5-min intervals (Portavo 907 Multi pH, Knick, Berlin, Germany). The acidification was stopped at pH 4.8, and the fermentation time was used as a descriptor to calculate the acidification activity of each starter. After acidification with S, SL or SLB, the obtained suspensions were centrifuged (10,000× *g*, 30 min, 4 °C). Pellet samples (P) (PS, PSL, PSLB), containing mainly globulins and biomass, and supernatants (A) (AS, ASL, ASLB), containing mainly albumins, were obtained, respectively. In order to obtain the corresponding globulin-rich fractions (G) (GS, GSL, GSLB), respective pellet samples were solubilized in water (1:5 ratio), and the pH was adjusted to 7.5 with NaOH 0.5 M. The samples were kept under stirring overnight at 4 °C, and the pH was readjusted to 7.5. The solution was then centrifuged (10,000× *g*, 20 min, 20 °C), and the supernatant collected. The samples were stored in a freezer at −18 °C until utilization. Only one part of each albumin fraction was freeze-dried (Heto PowerDry PL6000, Thermo Scientific, Waltham, MA, USA) to prepare samples for differential scanning calorimetry analysis and nitrogen content. The freeze-drying condition were the same as the indicated by Oliete et al. [40]. As controls, pea protein fractions were obtained with conventional acid precipitation by using hydrochloric acid (HCl) 0.5 M or lactic acid (LA) 0.5 M following the same other steps. The different fractions, including pellets (PHCl, PLA), globulins (GHCl, GLA) and albumins (AHCl, ALA), were then compared with the fractions obtained by fermentation.

### 2.3. Proximate Analysis

Total nitrogen content (on dry basis) of obtained fractions was determined by the Kjeldhal method according to the AOAC International method 920.87 [41]. Approximately 10–200 mg (db) of protein solutions were hydrolyzed with 10 mL of concentrated sulfuric acid (96%) and one copper catalyst tablet (Kjeltab copper mini, BÜCHI AG, Flawil, Switzerland) and glass balls, in a digestion unit (SpeedDigester K-435, BÜCHI AG, Flawil, Switzerland) at 400 °C for 2 h. After cooling, 25 mL of distilled water plus 1 droplet of phenolphthalein was added to the hydrolysates before neutralization and titration. The distillation and boric acid titration were performed with the BÜCHI distillation unit K-350/K-355. Released ammonia was absorbed in solutions of 2% boric acid, and nitrogen contents were determined by titration with 0.1 M HCl. The amount of total nitrogen was then calculated from Equation (1) in which V was the volume of hydrochloric acid used during the titration of samples, N was the normality of HCl, and m was the weight of the samples.

Total nitrogen (%) = (N × V(L) × 14)/m × 100(1)

The content in non-protein nitrogen was obtained following the method of Awolumate [42] with a small variation. An amount of 10–200 mg of protein samples were mixed with TCA 12% (1:5 ratio). The samples were kept under stirring for 15 min, and then, the supernatants were filtered. The content of nitrogen in the supernatant was measured as explained for total nitrogen. Finally, the amount of protein nitrogen was obtained by subtraction of non-protein nitrogen from total nitrogen.

Moisture content was determined according to AOAC 923.03 [41]. Each sample (~1 g) was weighted precisely in an aluminum plate and placed into oven at 105 °C until constant weight. Then, it was cooled down in a desiccator for 2 h. The dry mass was averaged between three measurements.

### 2.4. Nitrogen Extraction Yield

The nitrogen extraction yield was obtained according to Equation (2), where m_Nf_ was the mass of extracted nitrogen recovered in every extraction step (G, A), and m_Ni_ was the mass of the total solubilized nitrogen recovered from pea flour after alkaline solubilization at pH 7.5. These masses were calculated from the recovered total mass of each fraction multiplied by their respective nitrogen content determined by Kjeldhal method.

Extraction yield (%) = (m_Nf_/m_Ni_) × 100 (2)

### 2.5. Protein Composition by SDS PolyAcrylamide Gel Electrophoresis (SDS-PAGE)

The polypeptide composition of albumin and globulin fractions was characterized by SDS-PAGE for all the extraction conditions. Novex^TM^ electrophoresis gels at 10 to 20% Tris-Glycine were used. Samples were diluted at least half in sample buffer: 187.5 mM Tris-HCl, pH 8.9, 10% (*w*/*v*) glycerol, 2% (*w*/*v*) SDS and 0.05% (*w*/*v*) bromophenol blue, in the presence (reducing conditions) or absence (non-reducing conditions) of 2% (*w*/*v*) dithiothreitol (DTT). The samples under reducing conditions were heated in a water bath for 10 min at 95 °C. All the samples were prepared and then deposited in the wells of the gel to have 10 µg of protein per well (measured by Kjeldhal method with a nitrogen conversion factor of 5.25 [43]). Molecular weight protein marker from Sigma–Aldrich (SigmaMarker^TM^ S8445, wide range, Mw 6.5 to 200 kDa) was used. The migration was carried out at 35 mA per gel, with the following migration buffer: 0.3% (*w*/*v*) trizma base, 1.45% (*w*/*v*) glycine and 0.1% (*w*/*v*) SDS, in a Scientific^R^ Mini Gel Tank of Migration (Thermo Fisher Scientific Inc., Massachusetts, United States). The gels were then rinsed with distilled water, and the fixation was performed in four successive distilled water baths heated for 1 min in a microwave at 550 W. The staining of the gels was performed with Coomassie blue, Thermo Scientific^TM^ PageBlue^TM^ Protein Staining Solution, overnight. The discoloring was then achieved in several baths of distilled water, until the desired color.

### 2.6. Free Amino Groups

The content of free amino groups was measured with o-phthaldialdehyde (OPA) following the method of Church et al. [44]. The following compounds were diluted with water to 100 mL: 80 mg OPA (dissolved in 2 mL 95% ethanol); 50 mL 0.1 M sodium tetraborate buffer solution with pH 9.5; 5 mL 20% SDS; 0.2 mL of 2-mercaptoethanol. The OPA reagent was prepared immediately before use. Globulin and albumin fractions with a concentration of 10 mg protein/mL (measured by Kjeldhal method with a nitrogen conversion factor of 5.25 [43]) were prepared in phosphate buffer 0.1 M pH 7.0. An amount of 0.05 mL of the sample was added to 2 mL of OPA reagent. This solution was briefly stirred, and the absorbance at 340 nm was measured after a 2-min incubation period at room temperature. A standard curve was obtained by using leucine as a reference compound. Reference samples with a concentration ranging from 1 to 3 mM were prepared in phosphate buffer pH 7.0, and the leucine determination was performed as described above.

### 2.7. Differential Scanning Calorimetry (DSC)

Thermal properties of the albumin-rich and globulin-rich fractions were studied by DSC. Onset temperature (T_onset_), temperature of denaturation (T_d_) and enthalpy of denaturation (ΔH_d_) were determined using a MicroSC-4c microcalorimeter (SETARAM instrumentation, Caluire, France). Obtained protein solutions at 3% (*w*/*w*) (protein concentration measured by Kjeldhal method with nitrogen conversion factor of 5.25 [43]) were weighed in an aluminum pan, hermetically sealed and heated from 20 to 110 °C at 5 °C/min rate. Another pan filled with water served as reference. The experiments were repeated at least 3 times.

### 2.8. Statistical Analysis

One-way analysis of variance (ANOVA) was performed using Statistica software, version 12 (Tulsa, OK, USA). Tukey’s post hoc least significant differences method was used to describe means with 95% confidence intervals.

## 3. Results

### 3.1. Acidification Kinetics

**Figure 2:**
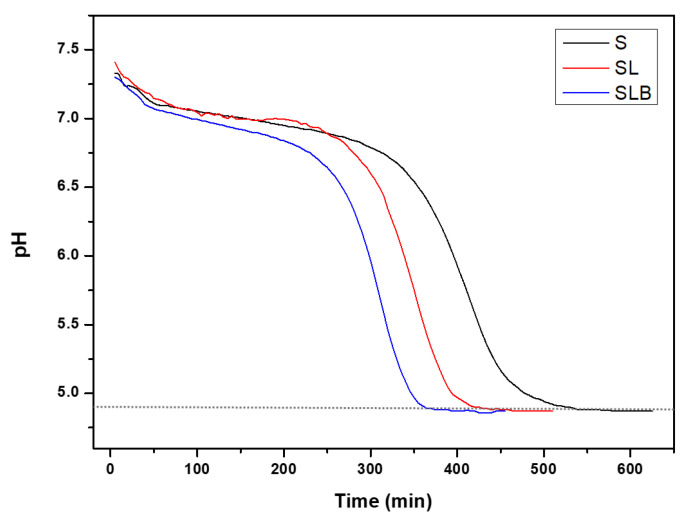
Acidification kinetics of bacteria (Streptococcus thermophilus (S), Streptococcus thermophilus + Lactobacillus acidophilus (SL), Streptococcus thermophilus + Lactobacillus acidophilus + Bifidobacterium lactis (SLB)) during the acid precipitation step of pea protein extraction.

Figure 2 shows the typical acidification kinetics of the cultures used (S, SL, SLB). Pea protein extract was a good substrate for the strains, as they were all able to reduce the pH up to 4.8. As pea seeds contain a part of fermentable sugars (𝛼-galactosides and sucrose) [45], it is likely that these sugars were also solubilized in the extracted protein suspension in a non-limiting amount for bacteria growth. It is well known that many LAB strains possess enzymes to metabolize these sugars into lactic acid [46]. Otherwise, the time required to reach the target pH was shorter for the mixed cultures. S showed the largest lag phase compared to the mixed cultures. It changed from lag phase to log phase in 5 h and then reached the stationary phase after 4 h. SLB had the highest growth rate in pea protein substrate. It took about 3 h for it to reach log phase and 2.5 h to reach the plateau. SL showed the same behavior as SLB, although it reached the stationary phase after approximately 3 h growth. Indeed, the acidification rate in lactic fermentation highly depends on the strains and the bacteria mixture [26,47]. For instance, fermentation of soymilk with different strains of Lactobacillus acidophilus and Bifidobacterium lactis revealed a sharp difference in the rate of acid production [48,49]. Furthermore, the synergistic effect of combined bacteria strains could cause an accelerated and efficient organic acid production during fermentation process itself, as already indicated [19]. The importance of such synergy on acidification activity of lactic acid bacteria has been studied extensively. Wang et al. [50] showed that lower pH and higher titrable acidity were obtained with mixed culture of Streptococcus and Bifidobacterium lactis compared to Streptococcus thermophilus alone. This was confirmed by De Souza et al. [51], who reported that the mixed cultures of Streptococcus thermophilus and Bifidobacterium lactis had better acidification profile than their mono-cultures. Helland et al. [52] also showed that Bifidobacterium lactis had better growth in co-culture fermentation with Lactobacillus acidophilus. Moon et al. [53] also compared the developed acidity of mixed and pure cultures. The authors reported that the growth of Streptococcus thermophilus increased in mixed culture with Lactobacillus acidophilus. Garcia et al. [54] showed the incorporation of Streptococcus thermophilus with lactobacilli strains led to higher acidification and significant reduction in the pH. Besides, Streptococcus thermophilus is always the dominant species in the mixed cultures, with the greatest growth regardless of the substrate [55].

### 3.2. Nitrogen Content in Albumin and Globulin Fractions

**Figure 3:**
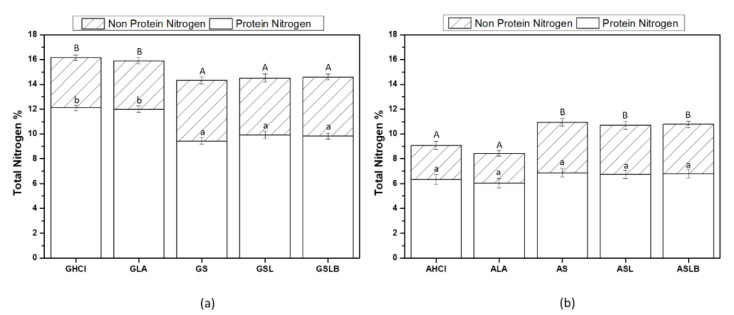
Total nitrogen (non-protein and protein nitrogen) content of (**a**) globulin fraction obtained by controls (GHCl, GLA) and fermentations (GS, GSL, GSLB), (**b**) albumin fraction obtained by controls (AHCl and ALA) and fermentations (AS, ASL, ASLB). Different capital letters represent significant differences in total nitrogen content, and small case letters represent significant differences in protein nitrogen among different samples (Tukey’s post hoc test).

Total nitrogen (protein + non-protein nitrogen) content of globulin and albumin fractions obtained by both lactic fermentations and controls is shown in Figure 3. As illustrated, globulin fractions obtained by controls (GHCl and GLA) showed higher total (~16%) and protein nitrogen content (~12%) than the fermented samples (GS, GSL, GSLB) (~14.5% and 9.5%, respectively). The majority of nitrogen was measured as protein nitrogen (Figure 3a). The non-protein nitrogen content for globulin fractions (~4%) did not differ significantly in all the samples, representing ~25–30% of the total nitrogen as already reported [56]. In the case of albumin fractions, the trends observed for total nitrogen or protein nitrogen between controls and fermented samples were reversed. Lactic fermented samples had higher values for both total (~11%) and protein nitrogen content (~7%), as well as non-protein nitrogen (~4%) compared to controls (~9%, 6% and 2%, respectively). However, this difference was significant only for total and non-protein nitrogen. Changes in nitrogen content of globulin and albumin factions when fermentation was used might be attributed to a combined effect of enzymatic proteolysis and acid-induced hydrolysis, implied by bacteria metabolism [28]. Indeed, during lactic fermentation, bacteria consumed part of the present nutrients for biomass production and eventually break the polypeptide chains to smaller polypeptides and amino acids [57]. During this metabolism, bacteria also produced organic acid (i.e., lactic acid) as a side product, which will interrupt the ionic interaction between side chains [35]. Changes in conformation and size of globulin would favor their solubility at pH 4.8, and thus, they would be released in the albumin-rich fraction. Furthermore, extensive hydrolysis of protein would produce amino acids that will increase the non-nitrogen content of the albumin-rich fraction. This could explain the increase in the total and non-protein nitrogen contents in albumin fractions for fermented samples compared to the controls and the decrease in total and protein nitrogen contents in globulin fractions, respectively. Increase in legume protein content by lactic fermentation was reported previously. However, previous authors did not study the changes in each fraction [34,35].

### 3.3. Nitrogen Extraction Yield

**Figure 4:**
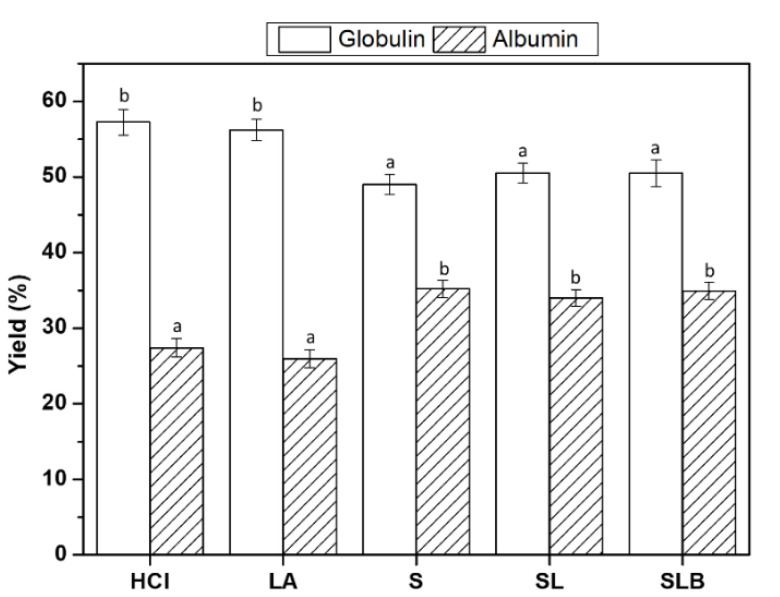
Nitrogen extraction yield of globulin and albumin fractions obtained by fermentations (S, SL, SLB) and controls (HCl, LA). Different letter represents significant differences among different samples (Turkey’s post hoc test).

The nitrogen extraction yields of all fractions was shown in Figure 4. Lactic fermented samples showed higher yields for albumin fraction and quite smaller for globulin one compare to the controls. These data confirmed the same trends as observed for nitrogen content in albumin and globulin fractions. Moreover, the results indicated no significant difference in extraction yield neither between controls (HCl and LA), nor between fermented samples. The sum of globulin and albumin fraction was inferior to 100% in all the samples, showing a reduction in yield of approximately 15%. It was probably the result of removing insoluble globulins and/or biomass after the final centrifugation applied to obtain the globulin suspension. For fermented samples, it could be hypothesized that biomass incorporated a significant amount of nitrogen, or more likely, that part of globulins was adsorbed at the surface of bacteria cells and was decanted simultaneously.

### 3.4. Free Amino Group Content

**Figure 5:**
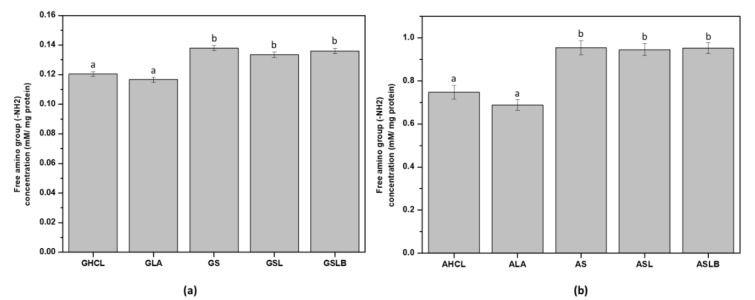
Free amino group content (mM/mg protein) of (**a**) globulin fractions obtained by controls (GHCl, GLA) and fermentations (GS, GSL, GSLB), (**b**) albumin fractions obtained by controls (AHCl, ALA) and fermentations (AS, ASL, ASLB). Different letter represents significant differences among different samples (Turkey’s post hoc test).

The content of the free amino group (-NH_2_) was determined by the standard method with OPA (Figure 5). All the fermented samples (for both albumin and globulin fractions) revealed higher amino group content compared to the controls. An increase in the content of the free amino group for fermented samples could be explained by the proteolytic activity of bacteria during fermentation probably releasing smaller polypeptides (<10 kDa), peptides and free amino acids [24,58,59,60,61]. Although the proteolytic activity and acid production capacity of the bacteria are known to be strain-dependent [58], no significant differences were observed between fermented samples in the present work. The value of the free amino group in albumin fraction (Figure 5b) was about tenfold higher for fermented samples compared to the globulin fractions, indicating that the smaller molecules produced were primarily present in albumin fraction as a soluble part. Free amino groups in the globulin fraction would correspond to those retained by insoluble protein and/or biomass when decreasing pH up to 4.8. The results obtained for the free amino group were consistent with the data measured for nitrogen content and extraction yield. An increase in the nitrogen content of albumin fractions in fermented samples could be explained by the enrichment of the extracts by soluble polypeptides and peptides originating from proteolysis. On the other hand, a decrease in nitrogen content in fermented globulin fractions could be attributed more likely to the elimination of some globulin polypeptides with the biomass.

### 3.5. Polypeptide Profile

**Figure 6:**
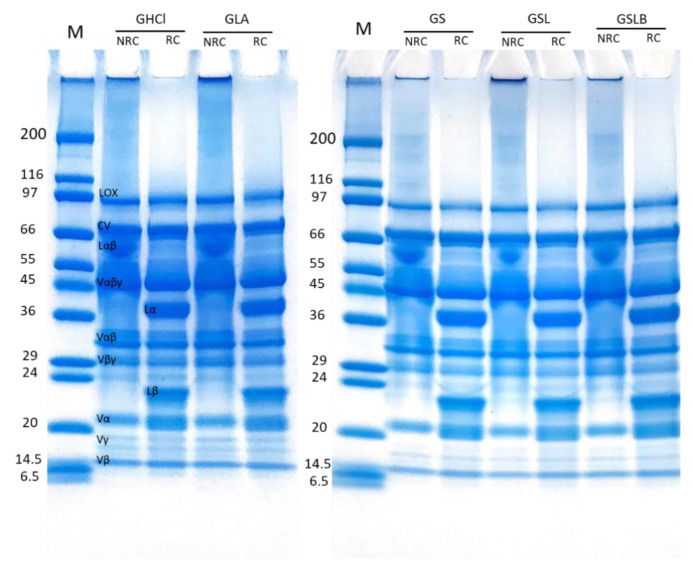
Electrophoretic profiles of globulin fractions obtained by controls (GHCl, GLA) and fermentations (GS, GSL, GSLB) in reducing (RC) and non-reducing (NRC) conditions.

**Figure 7:**
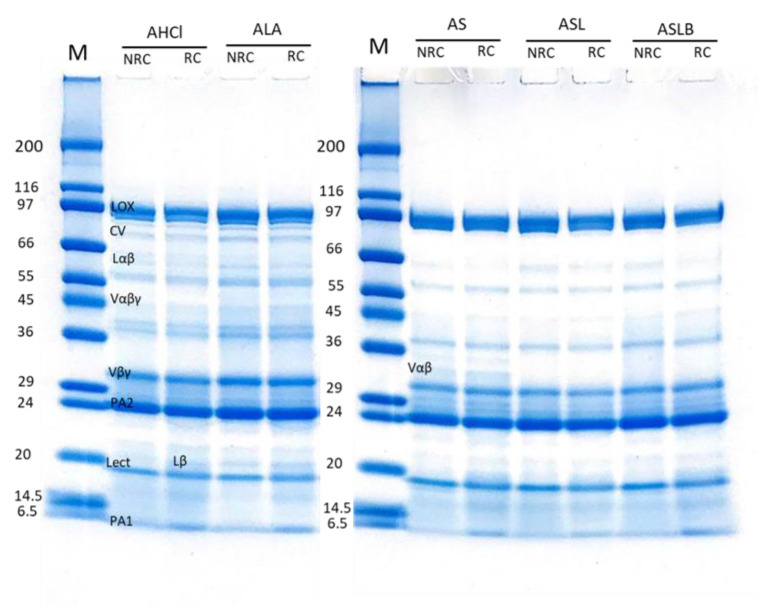
Electrophoretic profiles of albumin fractions obtained by controls (AHCl, ALA) and fermentations (AS, ASL, ASLB) in reducing (RC) and non-reducing (NRC) conditions.

SDS-PAGE was performed in non-reducing and reducing conditions to determine the effect of extraction method on polypeptide composition of the recovered globulin (Figure 6) and albumin (Figure 7) fractions. In non-reducing conditions (NRC), the globulin-rich samples’ profiles (Figure 6) revealed the presence of bands ranging from 10 to 99 kDa, characteristic of pea proteins [62]. The majority of these polypeptide bands represented various subunits of vicilin including the monomer (Vαβγ, ~50 kDa) and the cleavage-resulting polypeptides (Vαβ, ~30–36 kDa; Vβγ, ~25–30 kDa; Vα, ~20kDa; Vβ, ~13kDa; Vγ, ~12–16 kDa) [62,63]. There is also the presence of legumin monomer (Lαβ, ~60kDa), which separated to acid (Lα, ~40 kDa) and basic (Lβ, ~21–23 kDa) subunits in reducing condition [13]. The higher-molecular-weight bands corresponded to lipoxygenase (LOX, ~94 kDa) and convicilin (CV, ~71 kDa) [64]. The results indicated no significant difference in polypeptide profile of the globulin fractions obtained by either chemical acidification or lactic fermentation. The final polypeptide composition of globulin isolate was apparently not affected by the extraction method, although a global loss of globulins was observed, as indicated before. The presence of smaller polypeptides than 6 kDa, not observable on the electrophoretic profile because they migrated beyond the limit of the gel, cannot be excluded in the case of fermented samples, since free amino group content increased, as observed above.

Clearly absent in the previous patterns, the main 2S albumin subunits (PA2, ~26kDa; PA1, ~6kDa) [10,62] were observed in the electrophoretic profiles of albumin-rich samples, which also showed clear bands of LOX, lectine (Lect, ~17 kDa) [7] and some contaminations by globulin polypeptides, mainly Vαβ (Figure 7). The main polypeptides did not seem to be affected by the extraction method. Nevertheless, a few differences were observable for other contamination bands. The band of convicilin (~70 kDa) seemed to disappear completely in fermented samples. It might also be noticed that some bands (between 40–80 kDa) disappeared in fermented samples compared to the controls. Barkholt et al. [65] also claimed the disappearance of some high-molecular-weight bands in the protein profile of lactic-fermented pea flour compared to the non-fermented.

### 3.6. Thermal Properties

Thermal properties of native globulin-rich fractions were studied by differential scanning calorimetry (DSC). Onset temperature (T_onset_), temperature of denaturation (T_d_) and enthalpy of denaturation (ΔH_d_) of globulin fractions are presented on Table 1. Thermograms (data not shown) displayed one broad peak with a small shoulder, which according to literature, corresponded to the denaturation of 7S and 11S pea globulins, respectively [66]. T_onset_ for all the samples was approximately 61 °C. The first denaturation peak, with major surface, (T_d1_) had the highest value for GS (72.9 °C), followed by GLA (72.4 °C) and GSL (72.1 °C) samples. T_d1_ value for GS was significantly different from those evaluated for GHCl (71.7 °C) and GSLB (71.9 °C). The small differences observed between samples could be attributed to small variations in protein composition [67] and/or reflect the effect of ions added for acidification (chloride, lactate) or the metabolites produced during fermentation [68,69,70]. The minor denaturation peaks (T_d2_) corresponding to legumin did not vary significantly (~82–83 °C) between samples.

The most noticeable differences were observed in ΔH_d_, which was calculated from the total area of denaturation peaks. The enthalpy change reflects the extent of ordered structure of the globulins as the transition from native to denatured state took place [67]. ΔH_d_ of GS (15.11 J/g), GSL (14.66 J/g) and GSLB (14.76 J/g) were significantly higher compared to GHCl (12.57 J/g) and GLA (13.05 J/g). This value for GHCl was similar to alkaline-extracted pea globulin reported by Mession et al. [71]. Higher ΔH_d_ values in fermented samples could be representative of a higher-ordered structure of the protein in the globulin fractions obtained after fermentation. To explain these values, the vicilin/legumin area ratio was calculated from peak curve deconvolution (Table 1). The vicilin/legumin area ratio was lower in the fermented samples compared to the controls, indicating that more legumin was present in the case of fermented samples. As legumin has a more complex structure than vicilin, the extent of legumin denaturation energy was higher [71] leading to higher value for ΔH_d_. These results also indicated a relative depletion of vicilin and convicilin in the globulin-rich isolate meaning fermentation released more 7S globulins in the albumin fraction or some polypeptides from 7S globulins were consumed by bacteria, which was consistent with SDS-PAGE results.

Thermal properties of the albumin fractions obtained by both controls and lactic fermentations are shown on Table 2. The thermograms of albumin-rich samples showed two peaks (data not shown) at around 63 °C and 77 °C, respectively. Although there were no available data in literature regarding thermal denaturation temperature of pea albumins, this observation was in accordance with a previous study in our team [72]. The first peak considered as albumin denaturation was followed by a broad small peak, which was representative of the contamination by globulin polypeptides, corresponding to only around 1% of the total surface of both peaks. T_onset_ was close to 55 °C for all the samples. T_d1_ for controls (~62 °C) was slightly lower than fermented samples (~63 °C), which might reflect small differences in protein and co-solute composition. A similar trend was observed for ΔH_d_ among albumin fraction samples. ΔH_d_ values were significantly higher for fermented samples (~9.5 J/g protein) compared to controls (~8 J/g protein). This might be related to the improved protein purity in fermented samples as observed above. It could be also hypothesized that changes in albumins as a result of fermentation (chemical composition, conformation) exerted a better protective effect on protein structure upon thermal denaturation.

## 4. Conclusions

The pea protein extraction assisted by lactic fermentation successfully led to the production of globulin-rich and albumin-rich fractions. Compared to the common alkaline extraction–isoelectric precipitation method, fermentation led to an increase in total nitrogen content and protein yield of the albumin fraction. The increased protein solubility in the albumin-rich fraction was attributed to proteolytic activity of lactic acid bacteria, affecting probably some vicilin/convicilin polypeptides. As a consequence of fermentation effects, the recovery of protein in particular 7S globulins in the globulin-rich extract was decreased. Slight differences in thermal properties associated to the main pea proteins were observed, which could be explained by the changes in the composition of the protein fractions by fermentation. These findings are decisive for pea protein extraction in the food industry. Further studies need to be undertaken to better understand the impact of extraction assisted by different LAB strains on the functional, sensorial and nutritional properties of the isolated pea proteins. In particular, the identification of bioactive peptides released in the albumin fraction during fermentation would be useful for the valorization of enriched protein extract.

## Figures and Tables

**Table 1:** Thermal parameters of globulin fractions obtained by lactic fermentation (GS, GSL, GSLB) and controls (GHCl, GLA).

Sample	T_onset_ (°C)	Td_1_ (°C)	Td_2_ (°C)	ΔH_d_ Total (J/g Protein)	Peak 1/Peak2 Area Ratio
GHCl	61.58 ± 0.76 a ^1^	71.69± 0.3 a	83.29 ± 0.69 a	12.57 ± 0.24 a	3.5 ± 0.4 b
GLA	61.83 ± 0.18 a	72.43 ± 0.14 ab	83.05 ± 0.34 a	13.05 ± 0.26 a	3.5 ± 0.2 b
GS	61.93 ± 0.36 a	72.90 ± 0.04 b	83.31 ± 0.31 a	15.11 ± 0.40 b	2.2 ± 0.2 a
GSL	61.79 ± 0.06 a	72.15 ± 0.65 ab	81.97 ± 0.44 a	14.66 ± 0.33 b	2.6 ± 0.3 a
GSLB	61.84 ± 0.46 a	71.91 ± 0.45 a	82.23 ± 0.78 a	14.76 ± 0.17 b	2.4 ± 0.3 a

^1^ Different letters in the same column and type of sample represent significant differences among samples (Tukey’s post hoc test).

**Table 2:** Thermal parameters of albumin fractions obtained by lactic fermentation (AS, ASL, ASLB) and controls (AHCl, ALA).

Sample	T_onset_ (°C)	Td_1_ (°C)	Td_2_ (°C)	ΔH_d_ Total (J/g Protein)
AHCl	54.16 ± 0.24 a ^1^	62.14 ± 0.29 a	76.64 ± 0.18 a	7.87 ± 0.5 a
ALA	55.17 ± 0.89 a	62.21 ± 0.25 a	76.69 ± 0.04 a	8.36 ± 0.55 a
AS	54.11 ± 0.71 a	63.88 ± 0.13 b	78.56 ± 0.72 a	9.42 ± 0.49 b
ASL	55.70 ± 0.35 a	63.95 ± 0.16 b	78.15 ± 0.77 a	9.55 ± 0.54 b
ASLB	55.20 ± 0.47 a	63.71 ± 0.03 b	77.81 ± 0.38 a	9.43 ± 0.45 b

^1^ Different superscript in the same column and type of sample represent significant differences among different samples (Tukey’s post hoc test).

## Data Availability

Data sharing is not applicable to this article.
